# Early adversity causes sex-specific deficits in perforant pathway connectivity and contextual memory in adolescent mice

**DOI:** 10.1186/s13293-024-00616-0

**Published:** 2024-05-07

**Authors:** Rafiad Islam, Jordon D. White, Tanzil M. Arefin, Sameet Mehta, Xinran Liu, Baruh Polis, Lauryn Giuliano, Sahabuddin Ahmed, Christian Bowers, Jiangyang Zhang, Arie Kaffman

**Affiliations:** 1 Department of Psychiatry, Yale University School of Medicine, 300 George Street, Suite 901, New Haven, CT 06511 USA; 2 Bernard and Irene Schwartz Center for Biomedical Imaging, Department of Radiology, New York University School of Medicine, New York, NY 10016 USA; 3 Department of Biomedical Engineering, Center for Neurotechnology in Mental Health Research (CNMHR), The Pennsylvania State University, University Park, PA 16802 USA; 4 Yale Center for Genomic Analysis, P.O. Box 27386, West Haven, CT 06516-7386 USA; 5 Department of Cell Biology, Yale University School of Medicine, 333 Cedar Street, SHM IE26, New Haven, CT 06510 USA; 6 Center for Cellular and Molecular Imaging, Electron Microscopy Core Facility, Yale University School of Medicine, New Haven, CT USA

**Keywords:** Early adversity, Limited bedding and nesting, Perforant pathway, Myelination, Reelin, Cortical thinning

## Abstract

**Background:**

Early life adversity impairs hippocampal development and function across diverse species. While initial evidence indicated potential variations between males and females, further research is required to validate these observations and better understand the underlying mechanisms contributing to these sex differences. Furthermore, most of the preclinical work in rodents was performed in adult males, with only few studies examining sex differences during adolescence when such differences appear more pronounced. To address these concerns, we investigated the impact of limited bedding (LB), a mouse model of early adversity, on hippocampal development in prepubescent and adolescent male and female mice.

**Methods:**

RNA sequencing, confocal microscopy, and electron microscopy were used to evaluate the impact of LB and sex on hippocampal development in prepubescent postnatal day 17 (P17) mice. Additional studies were conducted on adolescent mice aged P29-36, which included contextual fear conditioning, retrograde tracing, and ex vivo diffusion magnetic resonance imaging (dMRI).

**Results:**

More severe deficits in axonal innervation and myelination were found in the perforant pathway of prepubescent and adolescent LB males compared to LB female littermates. These sex differences were due to a failure of reelin-positive neurons located in the lateral entorhinal cortex (LEC) to innervate the dorsal hippocampus via the perforant pathway in males, but not LB females, and were strongly correlated with deficits in contextual fear conditioning.

**Conclusions:**

LB impairs the capacity of reelin-positive cells located in the LEC to project and innervate the dorsal hippocampus in LB males but not female LB littermates. Given the critical role that these projections play in supporting normal hippocampal function, a failure to establish proper connectivity between the LEC and the dorsal hippocampus provides a compelling and novel mechanism to explain the more severe deficits in myelination and contextual freezing found in adolescent LB males.

**Supplementary Information:**

The online version contains supplementary material available at 10.1186/s13293-024-00616-0.

## Plain Language Summary

Childhood adversity, such as severe deprivation and neglect, leads to structural changes in human brain development that are associated with learning deficits and behavioral difficulties. Some of the most consistent findings in individuals exposed to childhood adversity are reduced hippocampal volume and abnormal hippocampal function. This is important because the hippocampus is necessary for learning and memory, and it plays a crucial role in depression and anxiety. Although initial studies suggested more pronounced hippocampal deficits in men, additional research is needed to confirm these findings and to elucidate the mechanisms responsible for these sex differences. We found that male and female mice exposed to early impoverishment and deprivation exhibit similar structural changes to those observed in deprived children. Interestingly, adolescent male mice, but not females, display severe deficits in their ability to freeze when placed back in a box where they were previously shocked. The ability to associate “shock/danger” with a “box/place” is referred to as contextual fear conditioning and requires normal connections between the entorhinal cortex and the hippocampus. We found that these connections did not form properly in male mice exposed to impoverished conditions, but they were only minimally affected in females. These findings appear to explain why exposure to impoverished conditions impairs contextual fear conditioning in male mice but not in female mice. Additional work is needed to determine whether similar sex-specific changes in these connections are also observed in adolescents exposed to neglect and deprivation.

**Supplementary Information:**

The online version contains supplementary material available at 10.1186/s13293-024-00616-0.

## Highlights

- The number of reelin-positive cells projecting from the lateral entorhinal cortex via the perforant pathway to the dorsal hippocampus is four times lower in adolescent male mice exposed to limited bedding (LB) compared to LB female littermates or control mice.
- Given the critical role that these projections play in hippocampal function, these findings provide a novel mechanism to explain the sex-specific deficits in contextual fear conditioning observed in adolescent LB male mice.
- Sparse axonal innervation in perforant pathway terminals also explains the reduced myelination observed in the stratum lacunosum moleculare, which is one of the most highly myelinated regions in the developing hippocampus.
- Adolescent LB male and female mice are hyperactive and exhibit significant cortical thinning, which mirrors findings in humans exposed to severe early deprivation.

**Supplementary Information:**

The online version contains supplementary material available at 10.1186/s13293-024-00616-0.

## Background

Early-life adversity (ELA) is a heterogeneous group of childhood experiences that include abuse, neglect, severe poverty, bullying, racism, and exposure to high levels of neighborhood violence [1]. Different adversities lead to somewhat distinct developmental outcomes [2–4], which are difficult to delineate because individuals are commonly exposed to multiple adversities [4–6]. ELA is now recognized as a potent risk factor for abnormal brain development and an increased risk for multiple psychiatric and medical conditions [2, 7]. Some of the most reproducible findings in individuals exposed to ELA are reduced hippocampal volume and abnormal hippocampal function [2, 8–11]. Although some evidence indicates more pronounced volumetric changes in men exposed to ELA [2], several key questions remain unanswered. For example, it is unclear if all, or only some subtypes of ELA (e.g., abuse, neglect, etc.), lead to different structural and functional outcomes in the hippocampus of men and women. The cellular and molecular mechanisms responsible for these sex differences and their contribution to hippocampal function and psychopathology also need to be clarified. We currently do not know whether sex differences in hippocampal function are more pronounced in childhood, adolescence, or adulthood. This is an important question, especially considering that sex differences in psychopathology and task-mediated hippocampal activation seem to be more pronounced in adolescent males [1, 9, 12].

These are difficult questions to address in the clinical setting due to the inherent heterogeneity and complexity of ELA, genetic variability, and lack of access to the developing human brain [1]. Moreover, establishing causal links between structural and functional changes in the hippocampus with alterations in cognition is difficult to achieve in humans. The conserved nature of hippocampal development across mammalian species [13–15], coupled with the observation that ELA impairs hippocampal development and long-term function across diverse species, including rodents [16], suggests that research in rodents may elucidate important details about the underlying biology. Indeed, elegant work in rodents has identified several mechanisms by which ELA, in the form of limited bedding (LB), impairs hippocampal function in rodents [17–19]. However, most studies to date have focused on outcomes in adult males, with only few examples examining this issue in male and female rodents [16, 20, 21]. Naninck et al. [20], reported more significant hippocampus-dependent deficits in adult males than in females. They attributed these differences to reduced levels of adult neurogenesis in LB males, but others have not replicated these findings [21, 22]. The only study examining the impact of LB on hippocampal function in adolescent male and female LB mice confirmed more severe deficits in males. However, the mechanisms responsible for these sex differences have not been clarified [21].

The commonly used LB paradigm occurs from postnatal day 2 (P2) to P9 [23]. This period corresponds to developmental processes in the human hippocampus that extend from the third trimester to approximately 1–3 years of age, precluding key developmental processes seen during childhood and adolescence. These include dentate gyrus maturation, synaptogenesis, dendritic arborization, synaptic pruning, GABAergic input, and myelination [24–27]. Therefore, extending the duration of deprivation will enable a more comprehensive assessment of sexually dimorphic developmental processes occurring during childhood and particularly in adolescence [28, 29]. Finally, the work to date has focused on identifying structural and functional changes within the hippocampus without considering the impact of LB and sex on critical input from the entorhinal cortex into the hippocampus. These connections originate from reelin-positive cells in layer 2 of the lateral entorhinal cortex (LEC) and the medial entorhinal cortex (MEC) to form the perforant pathway, which is the primary input into granule cells in the dentate gyrus (DG) and, to a lesser extent, pyramidal neurons in the CA2 and CA3 subregions of the hippocampus [30]. Mature perforant pathway terminals are visible relatively early in development, around postnatal day 10 (P10) and P14 in mice and rhesus monkeys, respectively [31–33]. Functional connectivity between these terminals and granule cells in the DG undergoes significant changes during childhood and adolescence, such as myelination and expansion of synaptic input onto granule cells [27, 33]. LEC input targets newly formed neuroblasts, thereby playing an important role in guiding postnatal neurogenesis and pattern separation [34]. Disruption of perforant connectivity impairs episodic memory in rodents [30, 35–38] and children [37], underscoring an essential role for these connections in normal hippocampal development and function.

Here, we address these concerns by extending the LB procedure from P0 (birth) to P25 and testing the impact of this manipulation on hippocampal development in prepubescent (P17) and adolescent (P28-36) male and female mice. Adolescence is defined as a period of sexual maturation that starts at around P28, marked by increased expression of the neuropeptide kisspeptin, vaginal opening in females, and the onset of significant weight differences between males and females [39–43]. At around P60, mice reach sexual maturity and are considered young adults [39–43]. Hippocampal changes were investigated in prepubescent P17 mice because some aspects of hippocampal development are completed at this age (e.g., dentate gyrus formation) while other developmental processes are at their peak (e.g., synaptic pruning) or are just commencing (e.g., myelination, GABAergic innervation, and maturation of glutamatergic synapses) [24–27]. In contrast, most developmental processes in the hippocampus reach adult-like levels in P34-36 adolescent mice [44–46].

Using RNA-seq, stereology, high-resolution confocal microscopy, and electron microscopy we found that LB causes significant deficits in myelination in the hippocampus of 17-day-old prepubescent mice. Focusing on the stratum lacunosum moleculare (SLM), one of the most highly myelinated regions in the developing hippocampus, we demonstrate that decreased myelination results from reduced axonal innervation. These deficiencies were more prominent in prepubescent males and persisted into adolescence. High-resolution ex vivo diffusion magnetic resonance imaging (dMRI) in P29 adolescent mice revealed significant cortical atrophy and reduced hippocampal volume in both male and female mice. The volume of the entorhinal cortex and structural connectivity with the dorsal hippocampus were more prominently reduced in 29-day-old adolescent LB males compared to LB females. Using retrograde tracing, we found that LB impairs the ability of reelin-positive cells in the lateral entorhinal cortex (LEC) to project and connect with the dorsal hippocampus in 36-day-old adolescent LB male mice, a reduction that was not observed in female LB littermates. Finally, deficits in connectivity between the LEC and the dorsal hippocampus were strongly correlated with abnormal contextual fear conditioning. These results suggest that reduced connectivity between the LEC and the dorsal hippocampus contributes to abnormal contextual fear conditioning in adolescent LB male mice. Female LB littermates, on the other hand, are able to maintain normal connectivity and intact contextual fear conditioning.

## Methods

### Animals

BALB/cByj mice (Jackson laboratories, stock # 001026) were bred in-house and kept on a standard 12:12 h light–dark cycle (lights on at 7:00 AM), with food provided ad libitum. Temperature and humidity were held constant (23 ± 1 °C and 43% ± 2), and background noise in the room was kept at dB: 56.5. All studies were approved by the Institutional Animal Care and Use Committee (IACUC) at Yale University and were conducted in accordance with the recommendations of the NIH Guide for the Care and Use of Laboratory Animals.

### Limited bedding (LB)

The limited bedding (LB) procedure was performed as described previously [47, 48]. Briefly, breeding cages were set up using a 3:1 female-to-male harem in standard mouse Plexiglas cages with 2 cups of corncob bedding and no nesting material. Visibly pregnant dams were transferred to maternity cages containing 2 cups of corncob bedding with no nesting material and 3 chow pellets on the floor. At birth (P0), litters were culled to 5–8 pups and randomized to either control (CTL) or limited bedding (LB). CTL litters were provided with 500 cc of corncob bedding, 15 cc of soiled bedding from the birth cage, and one 5 × 5 cm nestlet from P0-25. LB litters were provided with 125 cc corncob, 15 cc of soiled bedding from the birth cage, and no nestlet from P0-25. Bedding was changed on P7, P14, and P21. All mice were weaned on P26 and housed with 2–3 same-sex and condition littermates per cage with 500 cc of corncob bedding, no nesting material, and 2–3 chow pellets on the floor.

### Tissue collection and experimental design

Behavioral studies were conducted between (9:30–12:30) and tissue was collected between (13:00–15:00) to minimize changes associated with circadian rhythm [47, 48]. Prepubescent mice (n = 117) were processed at P17 for RNA-seq, stereology, biochemical assessment of DNA, RNA and protein contents in the hippocampus, immunohistochemistry, and electron microscopy (Additional file 1: Fig S1A). Exploratory behavior in a group of P29 adolescent mice (n = 69) was examined using the open field test with a subset of the mice immediately perfused after the behavioral testing for dMRI studies (n = 24, Additional file 1: Fig S1B). A second group of adolescent mice was tested in the contextual fear conditioning at P31-33 (n = 66) and then perfused for immunohistochemistry at P34 (n = 24, Additional file 1: Fig S1C). Finally, a third group of adolescent mice was injected with the retrograde tracer CTB at P28, tested in the contextual fear conditioning at P33-34 (n = 46) and perfused to assess the number of reelin-positive and CTB-positive cells in the lateral entorhinal cortex at P35 (n = 38, Additional file 1: Fig S1D).

### Assessing RNA, DNA and protein contents in the P17 hippocampus

To characterize RNA, DNA, and protein content in the hippocampus, P17 pups were anesthetized, and the right and left hippocampi were dissected, snap-frozen in liquid nitrogen, and stored at – 80 °C until further processing (n = 8–9 mice per rearing and sex). The left hippocampus was homogenized on ice in lysis buffer from the AllPrep^®^ DNA/RNA/Protein Mini Kit (QIAGEN, Hilden, Germany). A sample from the lysate was used to quantify total DNA and RNA content using the appropriate Qubit^®^ Fluorometer kit (Cat. # Q32850 for DNA and Q32852 for RNA). The remaining homogenate was then processed according to the manufacturer’s instructions to purify RNA, DNA, and proteins. Samples were stored at -80 °C until further processing. Purified total protein was quantified using a Pierce^TM^ BCA Protein Assay Kit (Cat. # 23,227).

### RNA-seq

RNA samples from the left hippocampus of P17 pups (CTL males = 7, CTL females = 8, LB males = 8, LB females = 8 from 4–6 independent litters) were sequenced at the Yale Center for Genomic Analysis. RNA quality and concentration were assessed using the 2100 Bioanalyzer on an RNA 6000 Nano Assay (Agilent Technologies, Inc., all RINs > 9). Libraries were constructed by amplifying 500 ng of total RNA using 9 PCR cycles with the KAPA mRNA HyperPrep Kit (KAPA Biosystems). cDNA libraries were validated using the Bioanalyzer 2100 on a High Sensitivity DNA assay and quantified using the KAPA Library Quantification Kit for Illumina Platforms on a Roche Lightcycler 480. Sequencing was performed on an Illumina NovaSeq 6000 using the S4 XP workflow 2 × 100 with each library using 1.25% of the S4 lane. Reads were trimmed for quality using custom scripts with a minimum accepted length of 45 bases. The trimmed reads were then aligned to the mm10 reference mouse genome using gencode annotation [49] and HISAT2 software [50]. Transcripts per million (TPM) estimation was calculated using StringTie [50].

### Immunohistochemistry

Perfused brains were sectioned coronally using a VT1000S vibratome (Leica) to obtain 50-micron sections and sorted into 6 pools, each containing 16–18 slices spaced at 300-micron intervals that systematically span the entire rostral-caudal axis of the brain. No staining was seen when primary antibodies were omitted, and the specificity of all antibodies was previously established [51–54] and further confirmed using RNA-seq and electron microscopy. Tissue was blocked with TBST buffer for one hour at room temperature [1X Tris-buffer saline (Bio-Rad, cat#1,706,435), 0.3% Triton X100 (Sigma-Aldrich, Cas# 9036–19-5), and 10% Normal goat serum (Jackson ImmunoResearch Laboratories Inc. cat# 005–000-121, RRID: AB_23369900]. After blocking, free-floating sections were incubated overnight at room temperature with rabbit PDGFRα (D1E1E) XP^®^ antibodies (Cell Signaling, cat #3174, RRID:AB 2162345, 1:200), mouse anti-APC (Ab-7) (also known as anti CC-1, Millipore, cat #OP80-100UG, RRID:AB 2057371,1:100), and rat anti-MBP (Bio-Rad, cat # MCA409S, RRID:AB 325004, 1:500). Axonal staining and myelination in the stratum lacunosom moleculare (SLM) were assessed with rat anti-MBP (Bio-Rad, cat # MCA409S, RRID:AB 325004, 1:500), mouse anti-neurofilament H (NF-H) phosphorylated antibodies (BioLegend, Cat# 801,602, RRID:AB_2715851, 1:1000). The number of reelin-positive cells was assessed using a mouse anti-reelin antibody (Abcam, Cat # ab78540, RRID:AB 1603148, 1:400). The slices were then incubated with the appropriate fluorescently labeled goat secondary antibodies (Thermo Fisher, Cat #A-21422/ RRID:AB 141822, # A-11008/RRID:AB 143165, # A-21094/ RRID:AB 141553, all at 1:200) and mounted on glass slides with VECTASHIELD HardSet antifade mounting medium with DAPI (Vector laboratories Cat# 10,955).

### Stereology, cell counting, and image analysis

Stereological analysis was performed by tracing the borders of the granule cell layer of the dentate gyrus (DG) and the pyramidal cell layer of the Cornu Ammonis (CA) regions of the hippocampus under low magnification (-2.5x) using Stereo Investigator 10 software (MBF Bioscience), and volume was calculated using Cavalieri’s principle [55, 56] and normalized for body weight. To quantify PDGFRα and CC1 cell numbers and MBP intensity, a 450 × 250 µm square area containing the SLM was selected and analyzed using Imaris 10.0 (Bitplane, Oxford Instruments). The object selection spots model was used to analyze PDGFRα and CC1 with the cell diameter set to 6.5 µm for PDGFRα and 13.5 µm for CC1, and the Z diameter was kept as default. MBP and NF-H intensities were determined using the object selection surface model using a 0.720 µm threshold with the intensity max filter applied. Colocalization between MBP and NF-H was performed with the Object-Based-Colocalization model using the shortest distance to surfaces of 0.1 µm. Three slices were counted per animal and averaged to determine the number of PDGFRα- and CC1-positive cells, MBP intensity, and MBP/NF-H colocalization for each mouse. For counting CTB-positive cells, a 200 X 300 μm square area containing layers 2–3 of the LEC was cropped, and the number of cells was counted in Imaris using the Spots Model with XY diameter of 20 μm, default Z-diameter, and max intensity filter settings. Five slices were counted per animal and averaged to obtain the number of reelin-positive cells in the LEC for each animal.

### Electron microscopy

P17 male mice were perfused with cold PBS followed by 4% paraformaldehyde in PBS. Brains were then dissected out and immersed in a solution containing 2.5% glutaraldehyde and 2% paraformaldehyde in 0.1 M sodium cacodylate buffer (pH 7.4) and kept overnight at 4 °C. Two hundred micron-thick sections containing the dorsal hippocampus (Paxinos coordinates, bregma = − 1.34 mm to − 2.80 mm) were cut using a VT1000S vibratome (Leica) and postfixed with 1% OsO_4_ and 0.8% potassium ferricyanide at room temperature for one hr. Specimens were then *en bloc* stained with 2% aqueous uranyl acetate for 30 min, dehydrated in a graded series of ethanol to 100%, substituted with propylene oxide, and embedded in EMbed 812 resin. Sample blocks were polymerized in an oven at 60 °C overnight. To locate SLM, semithin sections (250 nm) were prepared and stained with a solution of toluidine blue and 1% sodium borate. TEM thin sections (60 nm) were obtained using a Leica ultramicrotome (UC7) and poststained with 2% uranyl acetate and lead citrate. Sections were examined with an FEI Tecnai transmission electron microscope at an accelerating voltage of 80 kV, and digital images were recorded with an Olympus Morada CCD camera and iTEM imaging software. The MyelTracer software [57] was used to determine axonal diameter and G-ratio. This software automatically converts various axonal shapes into perfect circles and calculates the G-ratio as the ratio between the inner axonal diameter and the diameter of the axon plus the myelin sheath that encases it. N = 20 axons per mouse and n = 3 male mice per group.

### Behavior

Open field test was done as previously described [47, 58]. Briefly, mice were allowed to explore a 40 × 40 cm arena (Maze Engineers, # 3201) illuminated at 60 l ×  for 5 min during which the distance traveled and the time spent in the inner 15 cm-area were measured using the EthoVision tracking system (XT 17, Noldus Information Technology). Contextual fear conditioning was performed as previously described [58], except that acoustic cue conditioning was not tested on the third day of contextual fear conditioning. Briefly, during the first day of training, mice were allowed to explore a Med Associates’ fear conditioning chamber (Cat # VFC-008) equipped with a grid floor in the presence of a 2% lemon scent for 300 s. After 300 s of free exploration, mice were exposed to 5 shock-tone pairing at variable inter-trial interval (30–180 s). These were presented as 30 s discontinued tones (7500 Hz, 80 dB) that co-terminated with a 1 s 0.65 mA foot shock. There was a 30 s period at the end of the session following the last pairing and freezing behavior recorded using Med Associates’ Video Freeze software. On the second day, mice were returned to the same box and contextual freezing was determined for 300 s in the absence of a tone or a shock.

### Ex vivo* dMRI*

P29 adolescent CTL and LB mice (N = 6 mice per rearing and sex, a total of 24 mice) were perfused and processed for high-resolution ex vivo dMRI using a 7-Tesla MR system equipped with a 4-channel cryogenic probe as described previously [58, 59]. Whole-brain voxel-based morphometric analysis was used to identify local volumetric changes affected by rearing, sex, and their interaction (2 × 2 ANOVA, FDR corrected, α = 0.1, p < 0.0105, cluster size > 25 voxels). Fiber tractography was used to assess structural connectivity between the entorhinal cortex and the dorsal hippocampus following a previously described methodology [58–60].

### Retrograde labeling

P29 mice were anesthetized with isoflurane (1–3%), and 50 nl of the retrograde tracer Alexa Fluor™ 555 cholera toxin subunit B (Thermo Fisher Scientific, Cat # C34776, 0.5% wt./vol in PBS) was injected at 10 nl/sec into the left SLM (ML: − 1.2, AP: − 2, DV: − 1.9) using a 5 µL Hamilton syringe equipped with a 32 blunt needle (Hamilton, Cat# 7803–04, 0.5 inch, PST 3). After injection, the needle was left in place for 5 min and then slowly removed. The incision was closed with Vet Bond, and the mice were allowed to fully recover prior to being returned to their home cage.

### Statistical analysis

Statistical analyses were performed using MATLAB R2022b (www.mathworks.com), SPSS (Version 29.0. Armonk, NY: IBM Corp.) and visualized with GraphPad Prism 9.0 (GraphPad Software, La Jolla California USA). The data were carefully screened for inaccuracies, with normality and homogeneity of variance confirmed using Kolomogrov-Smirnov and Mauchly’s tests, respectively. Outliers were removed if they were more than 2 standard deviations above or below the mean. Sample sizes were determined based on effect sizes obtained from preliminary studies, with alpha = 0.05, and a power > 0.8. Data were examined using a 2 × 2 ANOVA with rearing condition (CTL, LB) and sex as fixed factors. Significant rearing by sex interaction (e.g., p < 0.05) was followed by Sidak’s post hoc analysis for each sex. Significant main effect of rearing or interaction were followed by a preplanned quantification of Cohen’s simple effect sizes for each sex. RNA-seq analysis was conducted by first removing genes with low expression (average TPM < 3 in all three groups). This was done to increase statistical power and focus on genes that are more likely to drive developmental changes. Counts of the 13,245 genes that passed the initial screen were analyzed using DESeq2 script in R [61] to identify genes that were differentially expressed between CTL and LB using a Benjamini‒Hochberg false discovery rate (FDR) < 0.05. All raw data and analyses are available at GEO archive accession # GSE142305. Enrichment and transcription factor analyses were conducted using MetaCore™ (Clarivate Analytics) workflows on gene lists that passed a corrected p value of < 0.05.

## Results

### LB Inhibits oligodendrocyte progenitor-cell differentiation in the stratum lacunosom moleculare (SLM)

Consistent with previous studies [47, 48, 58], LB decreased body weight in P17 male and female pups (Additional file 1: Fig S2 A, B). Stereological analysis indicated that LB decreased the size of the granule cell layer (GCL) and the Cornu Ammonis (CA**)** pyramidal layer even after correction for body weight, with similar outcomes observed in males and females (Additional file 1: Fig S2C). The DNA, RNA, and protein contents extracted from the hippocampus were also reduced in male and female P17 LB pups (Additional file 1: Fig S2D). These findings replicate previous research demonstrating that LB reduces hippocampal volume in prepubescent pups [22, 47].

**Fig. 1:**
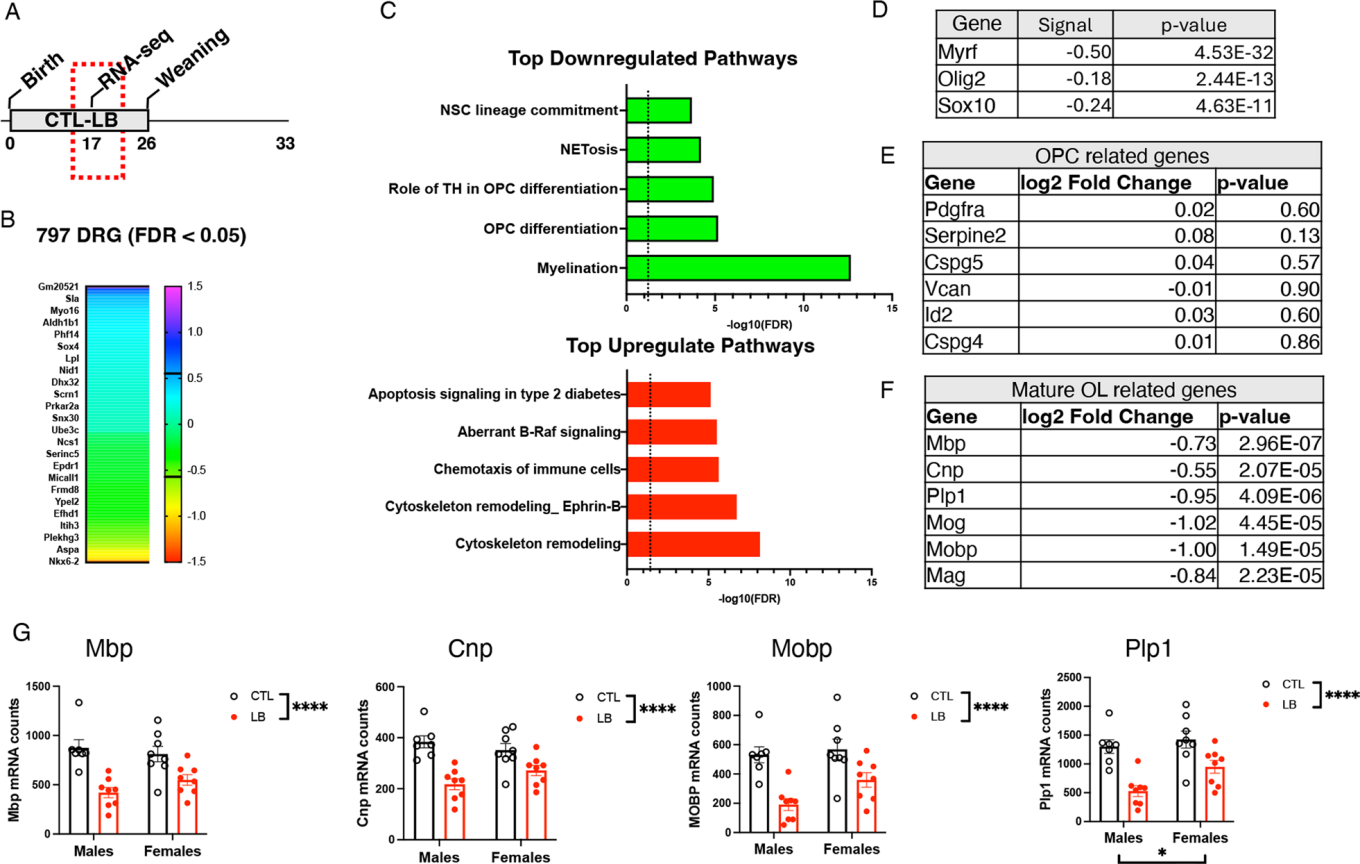
LB inhibits oligodendrocyte differentiation in the hippocampus of P17 prepubescent pups. **A** Experimental timeline. **B** Heatmap showing the effect size of differentially regulated genes (DRGs) at FDR < 0.05 between CTL and LB. **C** Pathway analysis of the top downregulated and upregulated pathways affected by LB. **D** Transcription factor analysis identified changes in Myrf, Olig2 and Sox10 activity as the most dysregulated factors affected by LB. LB does not affect the expression of OPC-specific genes **E** but significantly decreases the expression of genes expressed in mature oligodendrocytes (**F**–**G**). N = 7–8 mice per rearing and sex group. Error bars represent mean ± SEM. *p < 0.05, **p < 0.01, ****p < 0.0001

Bulk RNA-seq was used to further elucidate the mechanisms by which LB alters hippocampal development (Fig. 1A). Similar changes in gene expression were observed in male and female LB mice prompting us to focus on rearing-induced changes in gene expression. Using this approach, we identified 797 differentially regulated genes (DRGs) between CTL and LB (FDR < 0.05), most of which had a small effect size (i.e., log2 < 0.4, green/turquoise heatmap, Fig. 1B). Pathway analysis identified oligodendrocyte differentiation and myelination as the most significantly downregulated pathways in LB, whereas cytoskeleton remodeling was the most upregulated pathway in LB (Fig. 1C). The 20 most upregulated and downregulated genes are shown in Additional file 1: Fig S2. Transcription factor analysis identified reduced activity of Myrf, Olig2, and Sox10 as the key regulators of abnormal myelination in LB mice (Fig. 1D). Since Myrf is necessary for oligodendrocyte differentiation [53, 62], we assessed the effects of LB on genes that are specifically expressed in oligodendrocyte progenitor cells (OPCs) and mature oligodendrocytes (OLs). LB had no impact on OPC-specific genes (Fig. 1E) but significantly reduced the expression of OL-specific genes (Fig. 1F), indicating that LB impairs the differentiation of OPCs in the developing hippocampus. A 2 × 2 ANOVA examining the impact of rearing and sex on some of the most highly expressed OL-specific genes confirmed a highly significant effect of rearing (Fig. 1G). No significant effect of sex or interaction were found, except for Plp1 mRNA, where a significant effect of sex was observed. Nevertheless, a preplanned analyses of effect sizes found greater deficits in LB males (Fig. 1G. Cohen’s d effect sizes MBP: males = 2.45 vs females = 1.3, CNP: males = 2.71 vs females = 1.22, MOBP: males = 2.61 vs females = 1.21, Plp1: males = 2.66 vs females = 1.28).

**Fig. 2:**
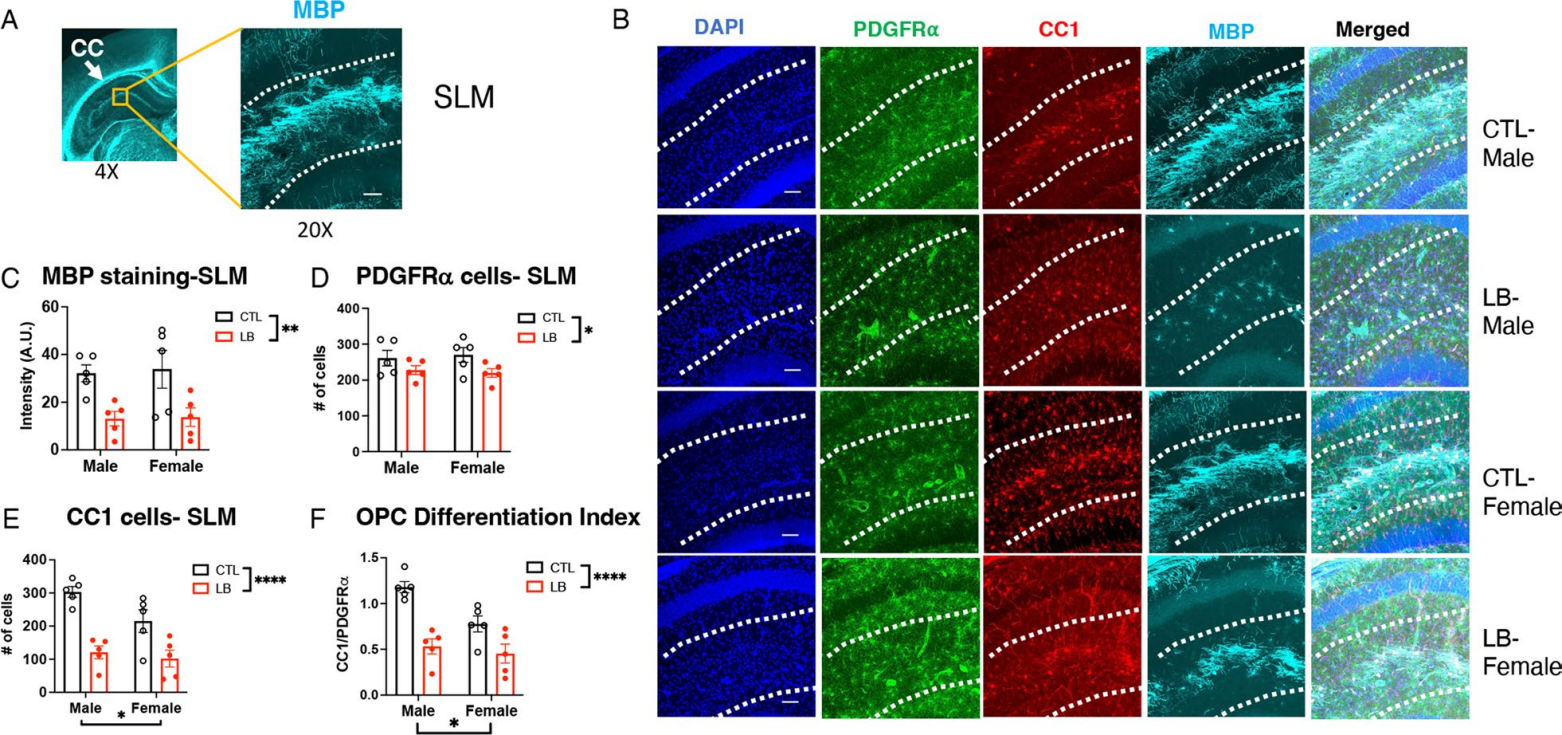
OPC differentiation in the SLM is impaired in P17 Prepubescent LB mice. **A** Low (4X) and higher (20X) magnification of myelin basic protein (MBP) staining in the SLM of P17 pups. **B** Representative images of PDGFRα-positive OPCs, CC1-positive mature oligodendrocytes, and MBP in the SLM. Quantification of MBP (**C**), PDGFRα-positive OPCs (**D**), CC1 mature oligodendrocytes (**E**) and OPC differentiation index (**F**) in the SLM group. N = 5–6 mice per rearing and sex group. Error bars represent mean ± SEM. *p < 0.05, **p < 0.01, ****p < 0.0001

To further validate the RNA-seq data, we assessed the effects of LB on oligodendrocyte maturation in the stratum lacunosum moleculare (SLM), one of the most myelinated regions in the developing hippocampus (Fig. 2A) [27, 63, 64]. This was done by quantifying levels of MBP as a marker of myelination, number of PDGFRα-positive cells (OPCs), and number of CC1 positive cells (OL) in the SLM of P17 pups. Consistent with the RNA-seq data, we found a significant reduction in MBP staining in LB compared to control (F (1, 16) = 15.42, P = 0.0012, η_p_^2^ = 0.49) with no significant effects of sex or interaction (Fig. 2B, C). There was also a significant reduction in the number of PDGFRα-positive cells in LB pups (Fig. 2B, D, rearing: F (1, 16) = 5.88, P = 0.0275, η_p_^2^ = 0.27, sex: F (1, 16) = 0.05, P = 0.8, interaction: F (1, 16) = 0.01, P = 0.91). For CC1-positive cells (i.e., mature-OL), there was a significant effect of rearing (F (1, 16) = 35.56, P < 0.0001, η_p_^2^ = 0.69) and sex (F (1, 16) = 4.664 P = 0.046). No significant interaction between rearing and sex was found but LB males showed a significantly greater reduction compared to LB female littermates (Cohen’s d effect size males = 4.58, females = 1.67, Fig. 2E). Since LB reduced both OPC and OL in the SLM, an OPC differentiation index was generated by dividing the number of CC1-positive cells by the number of PDGFRα-positive cells. There was a significant reduction in the OPC differentiation index in LB mice (F (1, 16) = 32.4, P < 0.000, η_p_^2^ = 0.67, Fig. 2F) indicating that impaired OPC differentiation is primarily responsible for the reduced myelination in the SLM. Again, no significant effect of sex or interaction were found, but males were more severely impacted compared to females (Cohen’s d effect size males = 3.99, females = 1.50, Fig. 2F).

### Axonal staining is reduced in the SLM of P17 LB mice

**Fig. 3:**
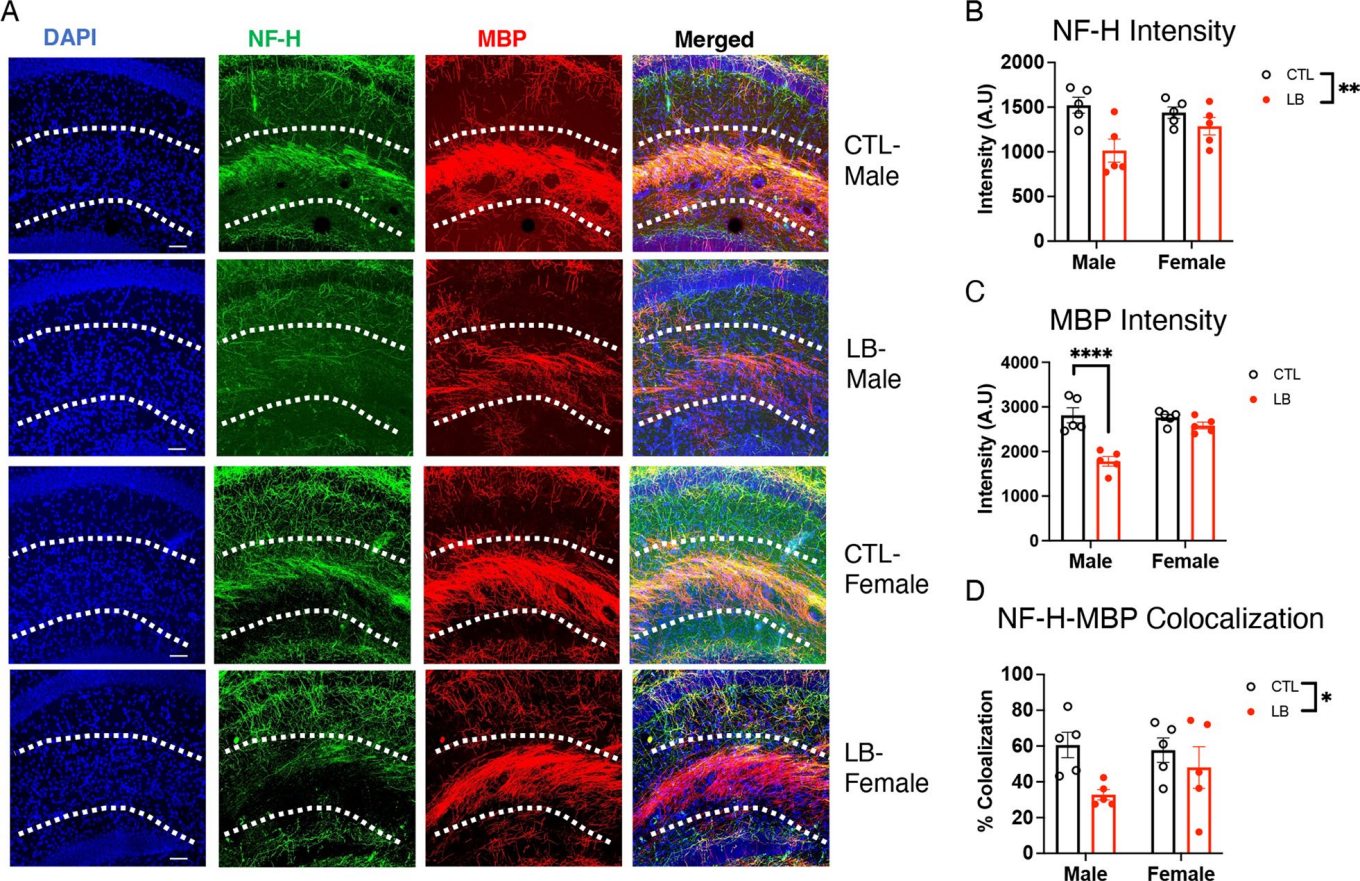
LB causes a more pronounced reduction in axonal staining in the SLM of Prepubescent 17-day-old LB male mice*.* **A** Representative images of axonal staining using anti-NF-H and anti-MBP antibodies in the SLM. Quantification of NF-H staining (**B**), MBP staining (**C**) and NF-H-MBP colocalization (**D**). Scale bars in Fig. 4A are 50 microns. N = 5 mice per rearing and sex. Error bars represent the mean ± SEM. *p < 0.05, **p < 0.01, ****p < 0.0001

To test whether the reduced myelination is due to abnormal axonal innervation, we probed the hippocampus with anti-phosphorylated neurofilament H antibodies (NF-H) to quantify axonal fibers in the SLM of P17 control and LB mice (Fig. 3A). There was a significant reduction in NF-H staining in LB mice (F (1, 16) = 11.38, P = 0.0039), with no significant effect of sex and a trend for rearing by sex interaction (F (1, 16) = 3.30, P = 0.088). The trend in interaction was due to a greater reduction in NF-H staining in LB males compared to LB females (Fig. 3B, Cohen’s d effect size males = 2.05, females = 0.82). A similar pattern was observed for MBP staining, with reduced myelination in LB males (P < 0.0001, Cohen’s d = 3.23) but not in females (P = 0.48, Cohen’s d = 1.11, interaction: F (1, 16) = 14.20, P = 0.0017, Fig. 3C). The percentage of neurofilaments co-stained with MBP was reduced in LB compared to CTL (F (1, 16) = 5.78, P = 0.029), with no significant effects of sex or interaction, but a larger impact in males compared to females (Cohen’s d effect size males = 2.28, females = 0.45, Fig. 3D). These results suggest that reduced axonal availability contributes to the abnormal myelination observed in the SLM of 17-day-old prepubescent LB mice, with males being more impacted than females.

**Fig. 4:**
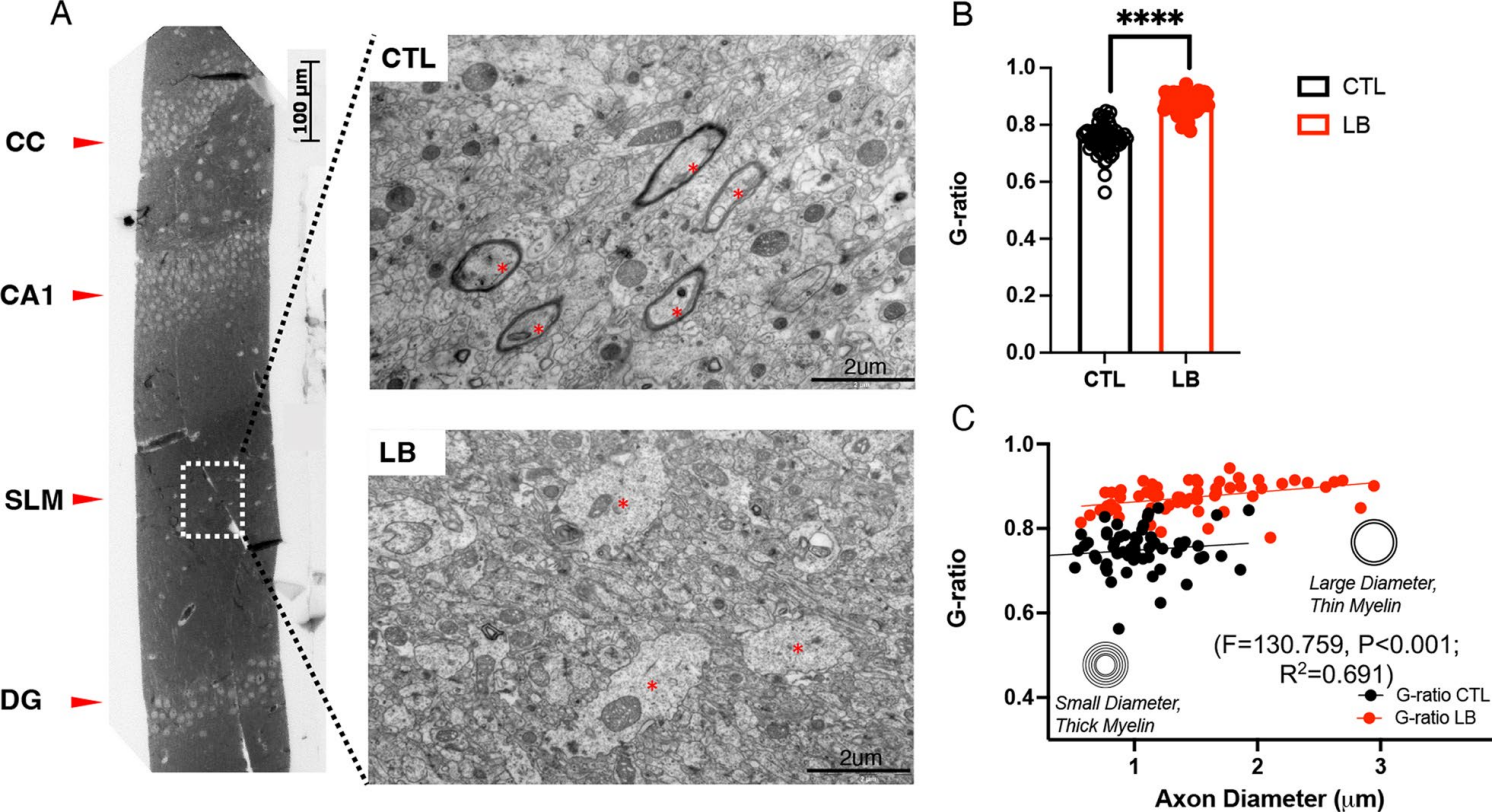
Transmission electron microscopy of myelinated axons in the SLM of P17 Prepubescent CTL and LB male mice. **A** Left, a light microscopy image of the same toluidine-stained sample to identify the SLM; Right, TEM images of the SLM in CTL and LB mice (visible axons indicated with red dots). **B** Image analysis of covariance between the G-ratio and axonal diameter (**C**). N = 20 axons per mouse with 3 male mice per condition. Error bars represent the mean ± SEM. ****p < 0.0001

Since LB prepubescent males show more pronounced deficits in myelination, we used electron microscopy to further characterize myelination in the SLM of P17 LB males. This investigation confirmed reduced axonal myelination in the SLM of 17-day-old LB males (Fig. 4A). LB males exhibited a significant increase in the G-ratio (t (118) = 15.57, P < 0.0001, Cohen's d = 2.84, Fig. 4B). Axonal diameter was also increased in LB compared to CTL males (CTL = 1.05 ± 0.04 µm, LB = 1.47 ± 0.074 µm, t (95) = 4.87, P < 0.001, Cohen's d = 0.89), indicating that the reduced myelination was not due to reduced axonal diameter. Furthermore, the increase in the G-ratio observed in LB remained significant even after adjusting for the increase in axonal diameter (ANCOVA, F = 130.759, P < 0.001, Fig. 4C). This indicates that the elevated G-ratio seen in LB males is mainly influenced by decreased myelination.

### Adolescent P29 LB mice are hyperactive and show extensive cortical atrophy

**Fig. 5:**
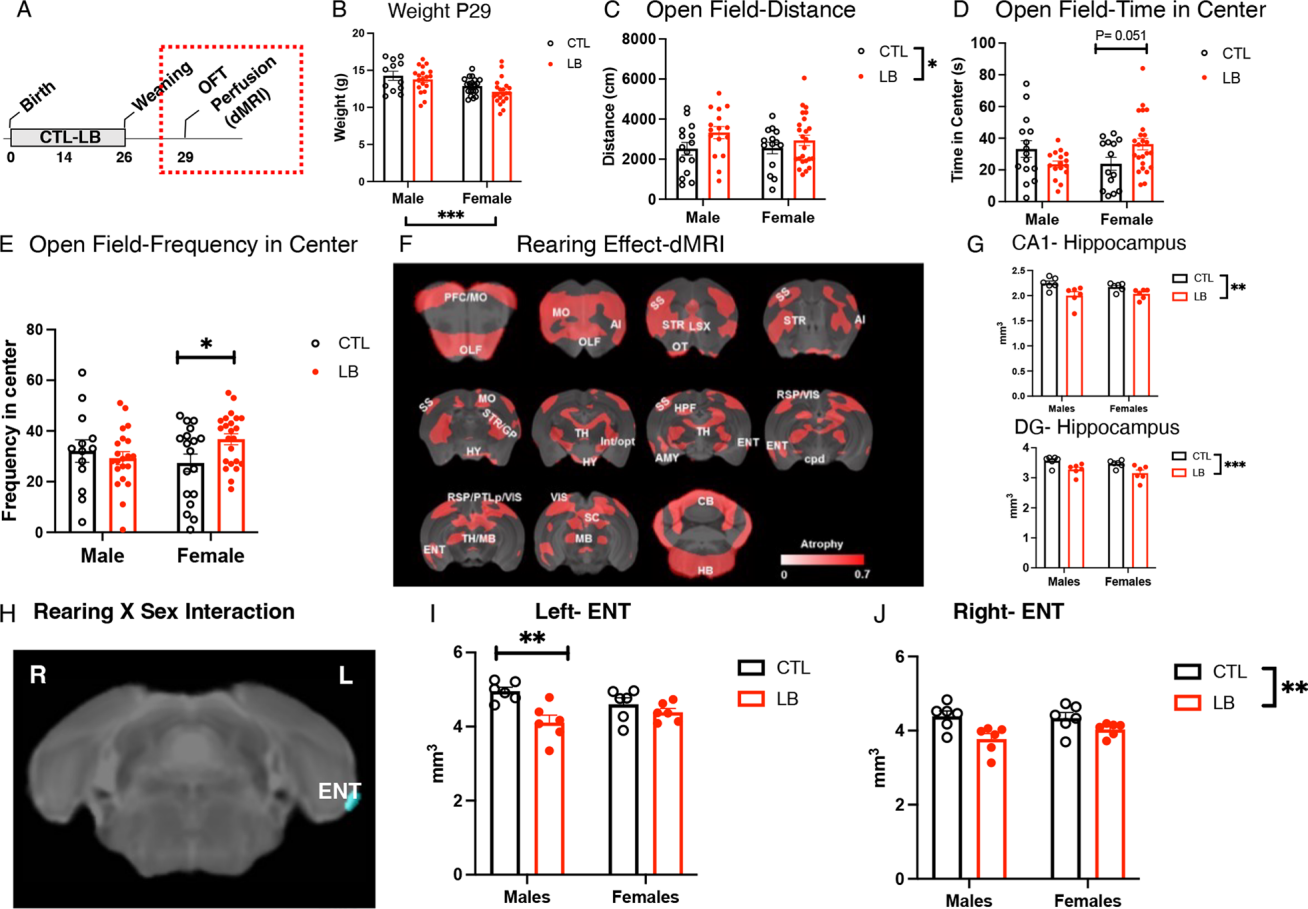
Adolescent P29 LB mice are hyperactive and show extensive reduction in cortical and subcortical gray matter. **A** Experimental timeline. Effects of rearing and sex on body weight (**B**), and exploratory behavior in the open filed test in P29 adolescent mice (**C**–**E**). CTL males N = 15, CTL females N = 14, LB males N = 16, LB females N = 24. **F** High resolution ex vivo dMRI found significant effects of rearing on volumetric changes using (minimal cluster size > 25 voxels, FDR < 0.1, p < 0.005). Areas with significant volume reduction are shown in red. N = 6 per rearing and sex. **G** Effects of rearing and sex on CA1 and dentate gyrus volumes. **H**–**J** The left entorhinal cortex was the only region showing a significant rearing by sex interaction (minimal cluster size > 5 voxels, FDR < 0.1, p < 0.0023). AI: Insular cortex, CB: Cerebellum, cpd: Cerebral peduncle, ENT: Entorhinal cortex, GP: Globus pallidus, HB: Hindbrain, HPF: Hippocampus, HY: HY: Hypothalamus, Int: Internal capsule, LSX: Lateral septal complex, MO: Motor cortex, OLF: Olfactory area, opt: Optic tract, OT: Olfactory tubercle, PFC: Prefrontal cortex, PTLp: Posterior parietal association cortex, RSP: Retrosplenial cortex, SC: Superior colliculus, SS: Somatosensory cortex, STR: Striatum, TH Thalamus, VIS: Visual cortex

We have recently proposed that LB is a mouse model of childhood deprivation and neglect [1, 48]. Given that hyperactivity and extensive cortical thinning are consistent findings in children and adolescents exposed to severe social and cognitive deprivation [65, 66], we used the open field test and high-resolution dMRI to evaluate these two outcomes in P29 adolescent male and female mice (Fig. 5A). A 2 × 2 ANOVA for body weight revealed a significant effect of sex (F (1, 67) = 14.46, P = 0.0003), with males being larger than females (Fig. 5B), consistent with previous research indicating that puberty initiated at around this age in mice [39–43]. No significant effects of rearing or interaction were observed for body weight at this age (Fig. 5B). LB male and female P29 adolescent mice showed a significant increase in total distance traveled in the open field test (F (1, 65) = 3.95, P = 0.05), with no significant effect of sex or interaction (Fig. 5C**)**. There was a significant interaction between rearing and sex for the time spent in the center (interaction: F (1, 65) = 7.71, P = 0.0072, Fig. 5D) and frequency of entering the center (interaction: F (1, 71) = 3.92, P = 0.05, Fig. 5E) due to an increased entry into the center in LB females but not LB males. These findings replicate our previous research in adolescent LB mice [47] and confirm increased hyperactivity in LB adolescent male and female mice.

Next, we conducted a 2 × 2 whole-brain voxel-based analysis to identify volumetric changes induced by rearing, sex, and their interaction [58, 59]. This unbiased approach revealed extensive volumetric reduction in LB adolescent male and female mice that included the sensory and motor cortex, prefrontal cortex, hippocampus, amygdala, and thalamus (shown in red in Fig. 5F). Follow-up region of interest analysis confirmed reduced CA1 (F (1, 20) = 15.25, P = 0.0009) and dentate gyrus volumes (F (1, 20) = 13.37, P = 0.0016) in both LB male and female P29 mice (Fig. 5G). These findings indicate that the lower hippocampal volume, seen in P17 LB pups (Additional file 1: Fig S2), persists in adolescent mice and is likely to represent global atrophy across multiple cortical and subcortical gray matter regions. Few brain regions showed a significant effect of sex (Additional file 1: Fig S4), and the only brain region that showed significant rearing by sex interaction was the left entorhinal cortex **(**Fig. 5H). A region of interest analysis confirmed a significant sex-by-rearing interaction in the left entorhinal cortex (F (1, 20) = 4.397, P = 0.049) driven by reduced volume in LB males (P = 0.0015, Cohen’s d = 2.18) but not LB females (P = 0.54, Cohen’s d = 0.61, Fig. 5I). A similar analysis in the right entorhinal cortex found a significant rearing effect (F (1, 20) = 11.85, P = 0.0026). No significant impact of sex (F (1, 20) = 0.61, P = 0.44) or interaction (F (1, 20) = 1.39, P = 0.25) were found, but the effect size in males was again larger (Cohen’s d effect size males = 1.72, females = 1.03, Fig. 5J).

### Reduced axonal innervation persists in P33 adolescent mice and correlates with deficits in contextual fear conditioning

**Fig. 6:**
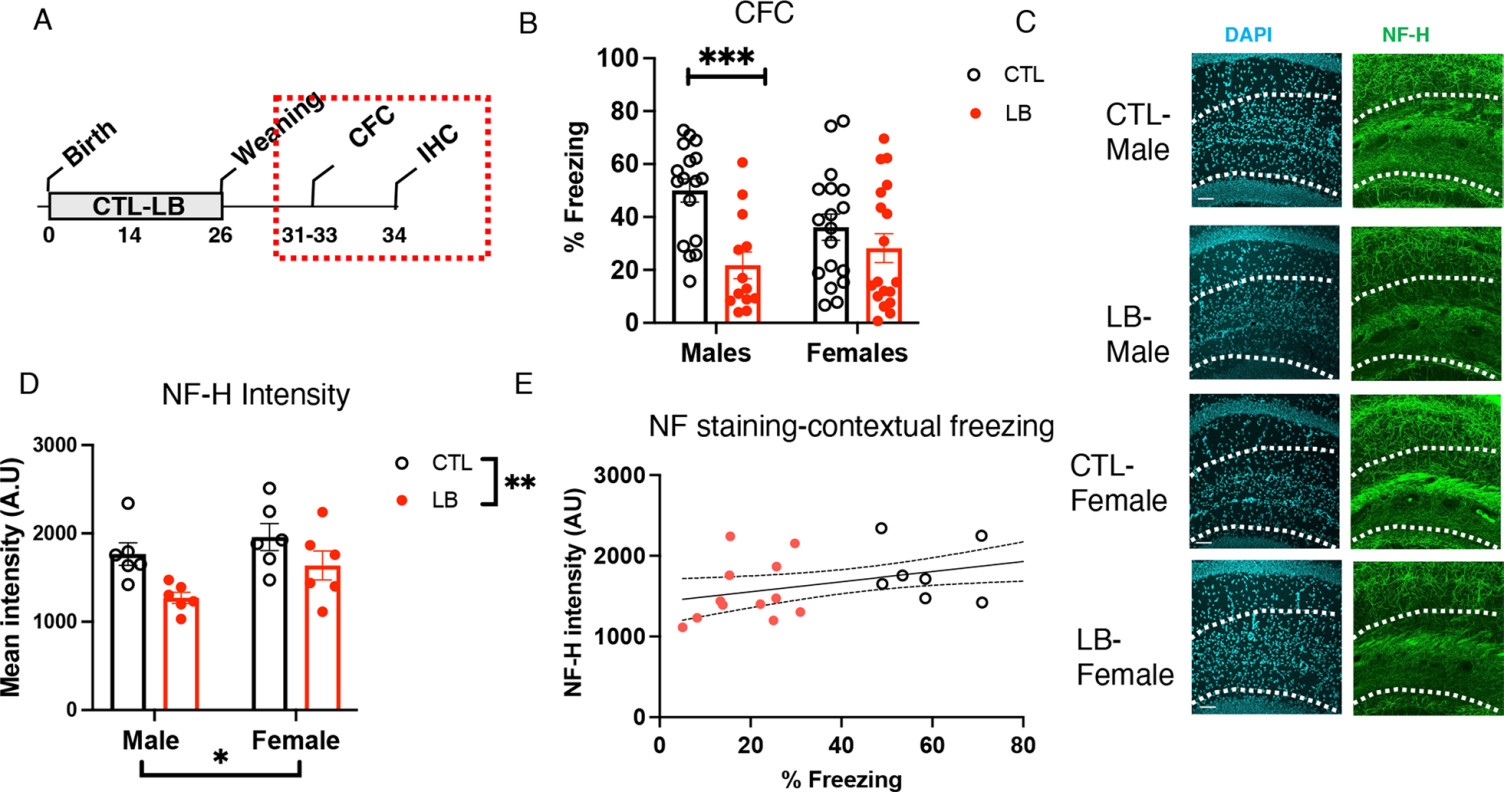
LB causes more severe deficits in contextual fear conditioning in adolescent P34 male mice. **A** Experimental timeline. **B** Contextual fear conditioning. CTL male = 17, CTL females = 18, LB males = 13, LB females = 18. **C**, **D** NF-H axonal staining in the SLM of adolescent mice, N = 6 mice per rearing and sex group. **E** The intensity of axonal staining correlates with contextual freezing behavior. Error bars represent the mean ± SEM. *p < 0.05, **p < 0.01, ***p < 0.001

Next, we tested whether the reduction in myelination and axonal staining seen in the SLM of P17 pups persisted and contributed to hippocampus-dependent deficits in adolescent mice. This was done by first testing contextual freezing in P31-33 adolescent mice and then assessing axonal staining and myelination in the SLM (Fig. 6A). Mice showed the expected increase in freezing in response to shock on the first day of training (F (2.958, 136.1) = 208, P < 0.0001). However, there was a significant interaction between rearing and sex for contextual freezing during the second day (F (1, 62) = 3.994, P = 0.05), driven by a significant reduction in freezing behavior in LB males (P = 0.0008, Cohen’s d = 1.55) but not LB females (P = 0.43, Cohen’s d = 0.36, Fig. 6B). Examination of NF-H staining in the SLM revealed significant effects of rearing (F (1, 20) = 9.55, P = 0.0058) and sex (F (1, 20) = 4.46, P = 0.047) but no significant interaction (F (1, 20) = 0.426, P = 0.52) (Fig. 6C). Nevertheless, as with the axonal staining at P17 (Fig. 4), the reduction in axonal staining was more pronounced in LB males (Cohen’s d = 2.01) than in LB females (Cohen’s d = 1.27), and the intensity of staining was significantly correlated with contextual freezing (Fig. 6E). No significant effects of rearing, sex, or interaction were observed for the number of PDGFR1α progenitor cells or CC1 mature oligodendrocytes in the SLM (Additional file 1: Fig S5A-C**)**. However, there was a significant reduction in MBP staining in the SLM of LB adolescent mice (F (1, 19) = 4.40, P = 0.049) that was again more pronounced in males compared to females (Additional file 1: Fig S5D). Together, these results indicate that reduced axonal staining and myelination persist in the SLM of adolescent LB mice and may contribute to the sex-specific deficits seen in contextual fear conditioning.

### LB reduces reelin-positive projections in male mice

**Fig. 7:**
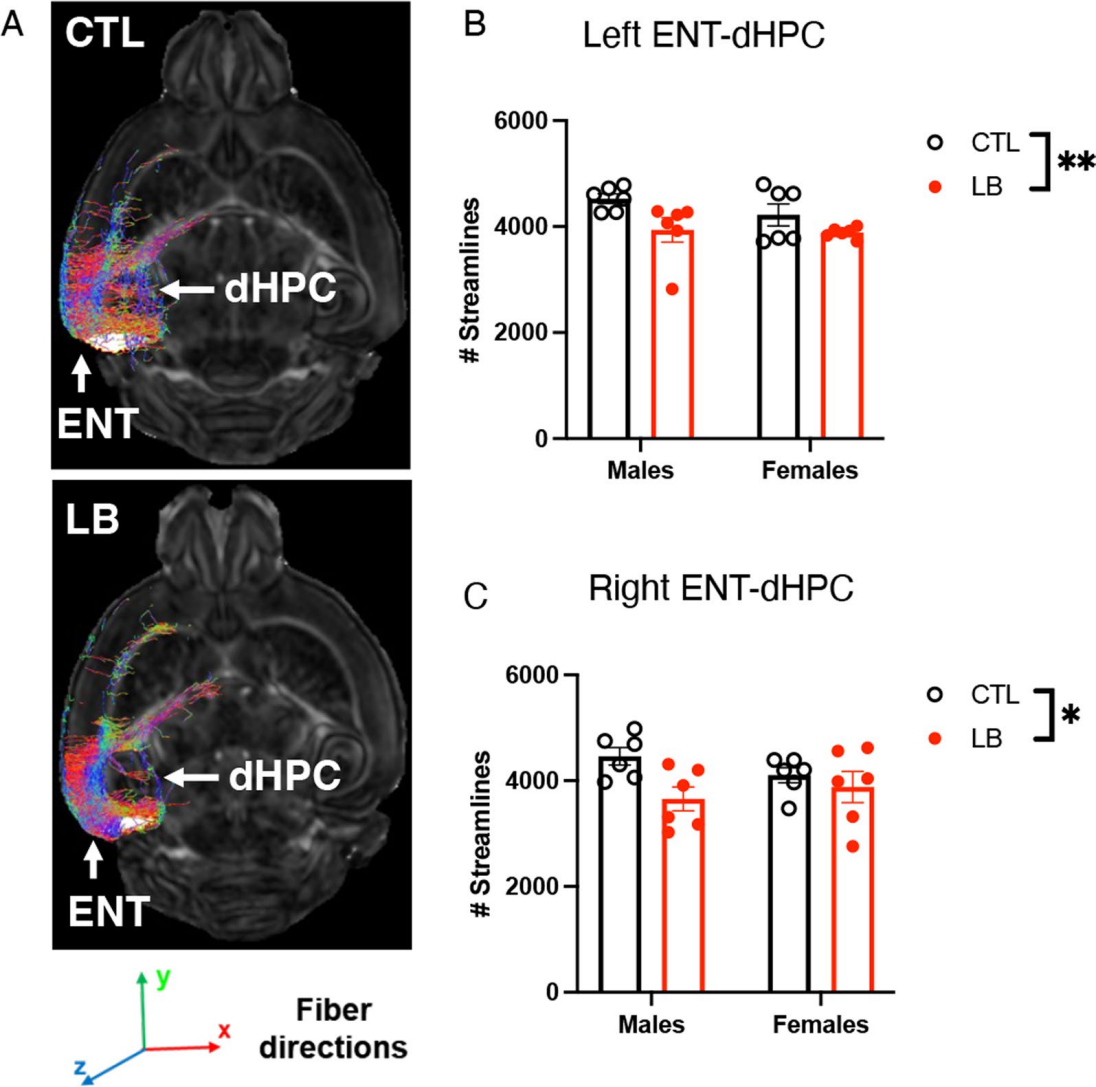
LB reduces structural connectivity between the entorhinal cortex (ENT) and the dorsal hippocampus (dHPC) in P29 mice. Representative images (**A**) and quantification of ENT-dHPC tractography in the left (**B**) and right (**C**) hemispheres. N = 6 mice per rearing and sex condition. Error bars represent the mean ± SEM. *p < 0.05, **p < 0.01

Most axonal terminals in the SLM originate from the entorhinal cortex [27], suggesting that reduced axonal staining in the SLM (Fig. 6D) reflects abnormal connectivity between these two brain regions in LB adolescent mice. Indeed, dMRI tractography revealed a significant reduction in structural connectivity between the entorhinal cortex and the dorsal hippocampus on the left (F (1, 20) = 8.241, P = 0.0095, Fig. 7A, B) and the right hemispheres (F (1, 20) = 5.659, P = 0.0274, Fig. 7C). Although there were no significant effects of sex or interaction between rearing and sex, the effect sizes in males were larger than those in females (Cohen’s d left: M = 1.39, F = 0.92; Cohen’s d right: M = 1.65, F = 0.39).

**Fig. 8:**
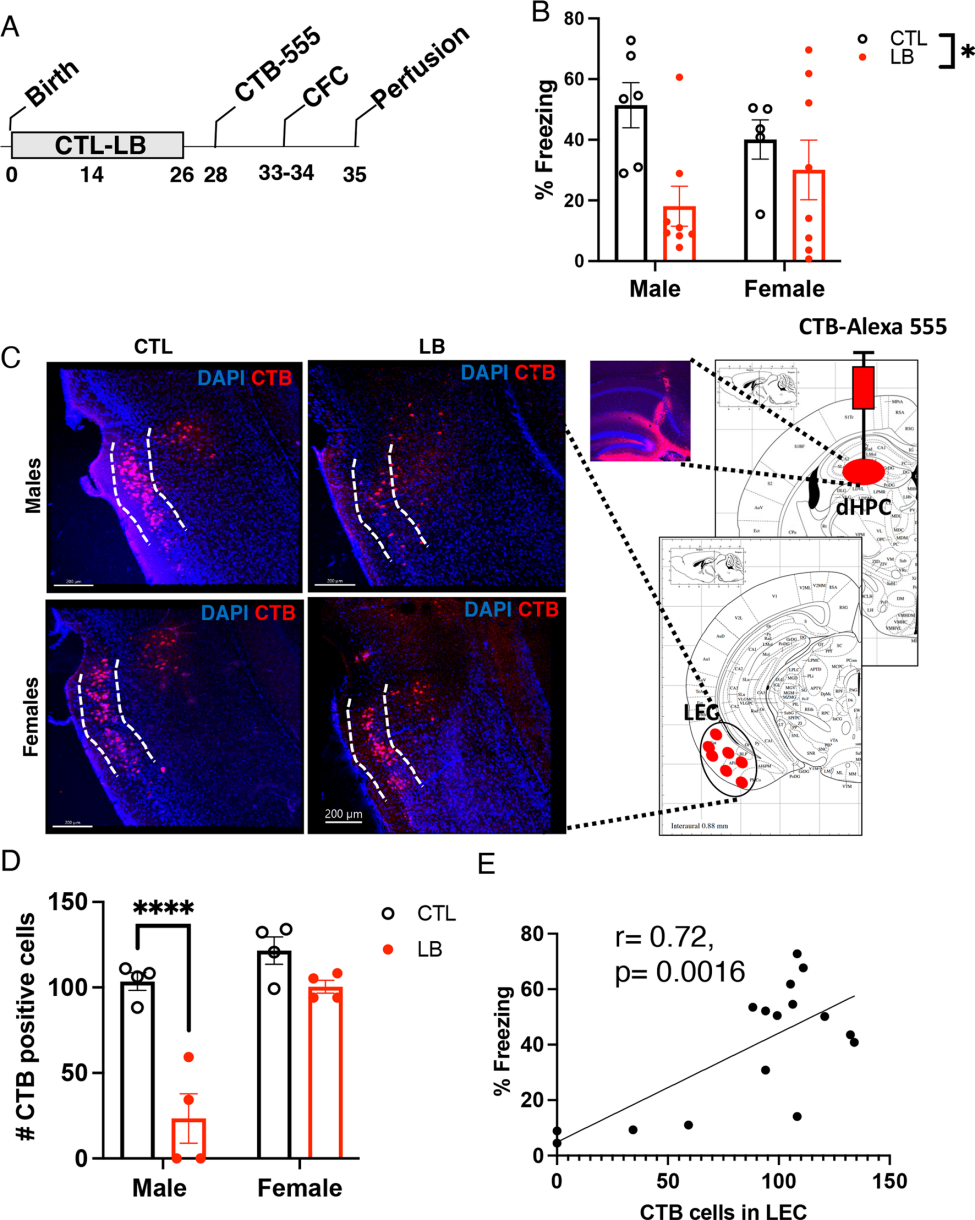
LB causes sex-specific deficits in lateral perforant pathway connectivity in P35 adolescent mice. **A** CTL and LB mice were injected with the retrograde tracer Alexa 555-CTB into the left SLM at P28, tested in contextual fear conditioning (P33-34), and perfused to assess the number of CTB-positive cells in the LEC at P35. **B** Contextual fear conditioning. **C** Schematic depiction of CTB injection at P28 and CTB labeling in the LEC at P35. **D** Number of CTB-positive cells in the LEC at P35. **E** The number of CTB-positive cells in the LEC correlates with contextual fear conditioning

To further validate these findings, we administered a fluorescently labeled retrograde tracer, Alexa-555-CTB, into the left SLM of P28 CTL and LB mice, tested contextual fear conditioning at P34, and processed the mice a day later to assess the number of CTB-positive cells in the left entorhinal cortex (Fig. 8A). LB mice showed a significant reduction in contextual freezing (F (1, 23) = 6.80, P = 0.016) that was more pronounced in males than in females (Fig. 8B). No significant effects of rearing, sex or interaction were observed for CTB labeling at the injection site (Additional file 1: Fig S6). However, there was a significant interaction between rearing and sex for the number of CTB-positive cells in the LEC (F (1, 12) = 11.08, P = 0.0060). This interaction was due to a fourfold reduction in the number of CTB-positive cells in LB males (P < 0.0001, Cohen’s d = 3.69) that was not observed in LB females (P = 0.22, Cohen’s d = 1.69, Fig. 8C, D). Moreover, the number of CTB-positive cells in the LEC was highly correlated with freezing behavior (Fig. 8E). Together, these findings indicate that LB reduces connectivity between the entorhinal cortex and the dorsal hippocampus in a manner that correlates with deficits in contextual memory and is more pronounced in adolescent males.

**Fig. 9:**
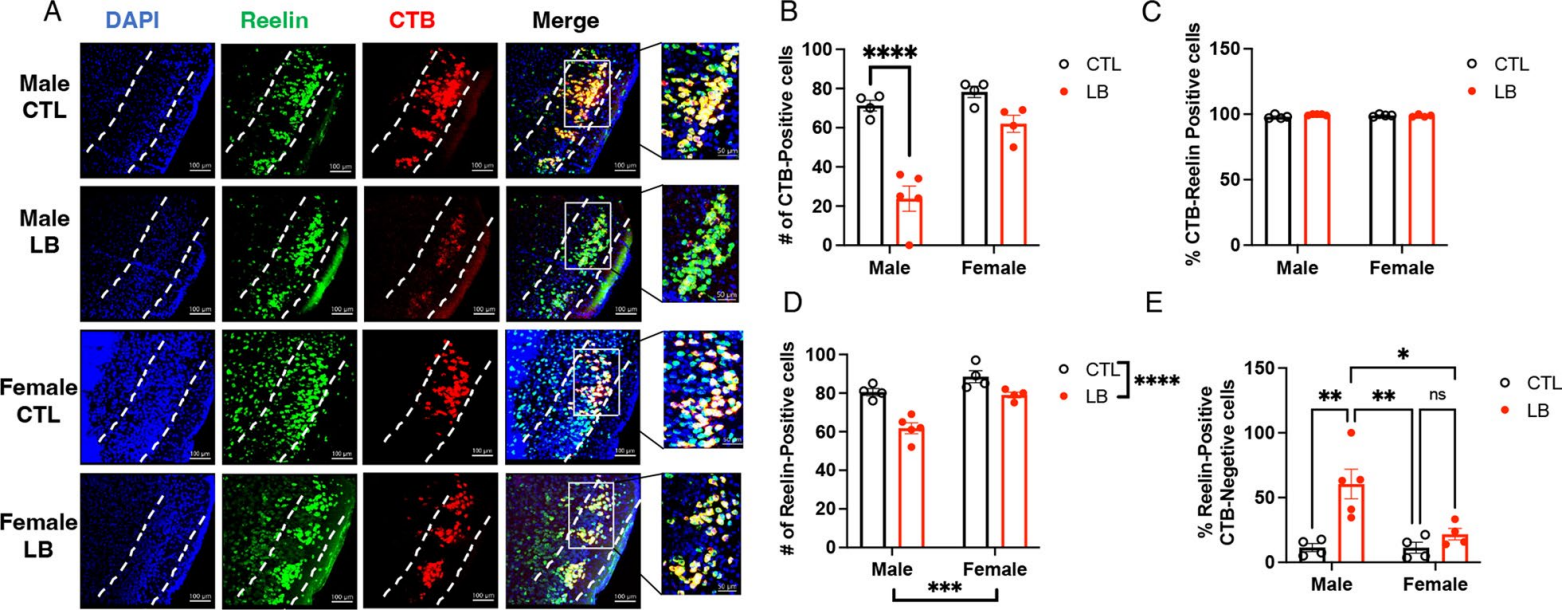
LB reduces the number of reelin-positive projections in adolescent males. **A** Representative images of Reelin- and CTB-positive cells in the LEC (scale bars in **A** are 50 µm). Higher magnification of rectangular areas of merged images is shown on the right. **B** The number of CTB-positive cells is reduced in the LEC of male LB mice. **C** All CTB-positive cells are reelin-positive regardless of rearing and sex conditions. **D** LB reduces the number of reelin-positive cells in the LEC. **E** Effects of rearing and sex on the percentage of reelin-positive CTB-negative cells in the LEC

Previous work has shown that reelin-positive cells project from the LEC to the dorsal hippocampus and that these projections are essential for contextual memory [30, 35, 36]. To determine whether the CTB-positive cells in the LEC were reelin-positive, we repeated the CTB retrograde labeling described in Fig. 8A using a second cohort of adolescent mice and replicated the sex-specific reduction in the number of CTB-positive cells in the LEC (Interaction: F (1, 13) = 10.76, P = 0.0060, Fig. 9A, B). We confirmed that all CTB-positive cells were also reelin-positive, regardless of rearing and sex (Fig. 9A, C), and found that the total number of reelin-positive cells in the LEC was reduced in LB mice, an effect that was more pronounced in males (rearing: F (1, 13) = 31.39, P < 0.0001, sex: (F (1, 13) = 25.50, P = 0.0002, interaction: (F (1, 13) = 3.399, P = 0.088, Fig. 9A, D). The percentage of reelin-positive and CTB-negative cells was significantly higher in LB males than in all other groups (interaction: F (1, 13) = 6.375, P = 0.025, Cohen’s d effect size males = 2.63, females = 1.19, Fig. 9A, E), indicating that most reelin-positive cells in the LEC fail to project to the dorsal hippocampus in LB males.

## Discussion

Clinical and preclinical studies have yielded evidence suggesting that exposure to ELA affects hippocampal development and function differently in males and females [1, 2, 9, 20, 21]. However, these findings necessitate additional replication and a more comprehensive exploration of potential underlying mechanisms. Consistent with previous work, we found robust deficits in contextual fear conditioning in adolescent males but not female mice. Exposure to LB reduced hippocampal volume in both males and females (Fig. 5F–G), suggesting that volumetric changes are unlikely to explain the sex differences in contextual freezing. In contrast, LB caused sex-specific volumetric changes in the entorhinal cortex and its connectivity with the dorsal hippocampus via the perforant pathway. More specifically, LB adolescent males showed a fourfold reduction in the number of reelin-positive projections to the dorsal hippocampus compared to CTL mice and LB females (Figs. 8D, 9B). Approximately 60% of the reelin-positive cells in the LEC of LB males fail to project to the dorsal hippocampus (Fig. 9E), suggesting that abnormal axonal pathfinding and/or axonal degeneration/retraction [67–69] are responsible for the reduced perforant pathway connectivity in LB male mice. The critical role that these projections play in contextual memory [30, 35–38] and the significant positive correlation between the number of these projections and contextual freezing suggest a compelling and novel mechanism to explain the more severe hippocampus-dependent cognitive deficits in LB adolescent males. More severe deficits in axonal staining and myelination were also observed in P17 prepubescent LB male pups (Figs. 1–3) suggesting that these sex differences emerge during the 2nd week of life when connectivity between the LEC and the dorsal hippocampus matures [27, 33]. Further studies are needed to clarify the exact age and the mechanisms by which LB impairs the formation of these connections. Additional studies are also needed to clarify the developmental impact of changes in connectivity on neurogenesis, synaptogenesis, and myelination and whether strategies that augment these connections can normalize contextual memories in LB male mice. Whether reduced perforant pathway connectivity alters synaptic maturation is of particular interest given recent work showing that LB reduced synaptic density and local functional connectivity in the dorsal hippocampus of adolescent males, but not LB female littermates [70].

### LB replicates key features of childhood deprivation in humans

Childhood neglect/deprivation is the most common form of early adversity [71, 72] leading to structural, behavioral, and cognitive deficits that are not typically seen in other forms of ELA, such as physical and sexual abuse [73–75]. Some key features of childhood neglect and deprivation include hyperactivity and cortical thinning [65, 66, 73–77], outcomes that have not yet been described in rodent models of ELA. The hyperactivity and extensive cortical atrophy observed in adolescent LB mice suggest that LB is a mouse model of early deprivation. These findings were seen in both males and females, indicating that sex-specific sequelae are not a global phenomenon but rather circuit specific.

Childhood neglect causes significant deficits in declarative memory [76, 77]. However, it is currently unclear whether these hippocampal deficits are more pronounced in adolescent males and whether they are associated with abnormal perforant pathway connectivity. Our ability to use imaging to quantify these connections and to assess their contribution to episodic memory in humans and mice provides an important strategy to validate findings across species. For example, exposure to traumatic brain injury in childhood disrupts entorhinal cortex-hippocampus connectivity and impairs episodic memory [37], demonstrating the feasibility of conducting similar studies in children exposed to early neglect and deprivation. Using high-resolution dMRI, we found a 10% reduction in structural connectivity between the entorhinal cortex and the dorsal hippocampus, with limited evidence for sex-specific effects (Fig. 7). In contrast, retrograde labeling revealed a 75% reduction in connectivity in LB males but not females (Figs. 8, 9), highlighting important differences in sensitivity between the two approaches. These differences likely reflect the lack of directionality and cell specificity associated with dMRI [78–80] and the need to further refine tractography tools in the mouse [58, 59, 81].

### LB impairs myelination in the SLM

Abnormal myelination is one of the most consistent findings in individuals exposed to early deprivation, with similar findings reported in nonhuman primates and rodents [82]. Only a few studies have examined the effects of LB on myelin development [47, 83], and the mechanisms responsible for these changes are yet to be clarified. Here, we show that LB inhibits OPC differentiation and myelination in the SLM of P17 prepubescent pups, with males being more impacted. This blockade is partly due to reduced axonal innervation, but the presence of hypomyelinated axons in the SLM of P17 males (Fig. 4) suggests that other factors also contributed to reduced myelination. Since myelination is driven by neuronal activation [82], it is possible that the impoverished conditions associated with LB cause abnormally low levels of neuronal activation in perforant pathway terminals that further impair OPC differentiation. In addition, recent work has shown that LB reduces the expression of sodium channels in OPC located in the developing hippocampus [83]. These channels may play a role in promoting OPC differentiation in response to neuronal activity [84–86]. Changes in microglial function may also contribute to the blockade of OPC differentiation. For example, LB reduces the expression of the receptor TREM2 in microglia [48], and low TREM2 levels are associated with abnormal OPC differentiation in a cuprizone model of remyelination [87]. LB also increases TNFα levels in microglia, which has been shown to inhibit OPC differentiation [88, 89]. The numbers of OPCs and mature oligodendrocytes in the SLM were similar in adolescent P34 CTL and LB mice indicating that the blockade of OPC differentiation is transient. Nevertheless, levels of MBP staining in the SLM continued to be lower in adolescent LB mice (Additional file 1: Fig S5), suggesting that deficits in myelination persist at this age. Whether hypomyelination reflects a reduction in axonal availability or other mechanisms are questions that remain unresolved. Additional studies are needed to clarify whether agents that augment myelination [82] could recover myelination in the SLM and improve contextual fear learning in LB mice.

## Perspectives and significance

This work bolsters earlier studies demonstrating that LB causes more pronounced hippocampus-dependent deficits in male compared to female mice. Importantly, we found that cognitive abnormalities are highly correlated with reduced connectivity between the LEC and the dorsal hippocampus. Given that these connections are essential for normal hippocampal function, these deficits in connectivity provide a novel and compelling mechanism to explain why males show more severe impairment in contextual fear conditioning. Adolescent LB male and female mice are hyperactive and have extensive cortical atrophy consistent with outcomes reported in children and adolescents exposed to severe early deprivation. These observations suggest that LB is a mouse model of early deprivation and that many developmental outcomes are similarly impacted in males and females. Elucidating the developmental principles by which ELA establishes sexual dimorphic changes in some circuits but not in others is an important area of investigation for future studies.

### Supplementary Information

Additional file1 (DOCX 9783 KB)

## Data Availability

RNA-seq raw data and analyses are available at GEO archive accession # GSE142305. All dMRI scans will be available at Figshare.
