# Supplementary material for: Early adversity causes sex-specific deficits in perforant pathway connectivity and contextual memory in adolescent mice
Source: Biol Sex Differ. 2024 May 7;15:39. doi: 10.1186/s13293-024-00616-0 (PMC11075329; doi:10.1186/s13293-024-00616-0)
Supplement: Supplementary file 1 — Additional file1 (DOCX 9783 KB) [file 13293_2024_616_MOESM1_ESM.docx]

**Additional File. Islam et al. BOSD 2024**

**Fig S1. Experimental Design and Timelines. (**A) Tissue was collected from prepubescent P17 mice for stereology, quantification of RNA, DNA, and protein contents in the hippocampus, RNA-seq, immunohistochemistry, and electron microscopy. (B) P29 adolescent mice were tested in the open field test and then immediately perfused for dMRI. (C) Adolescent mice were tested in contextual fear conditioning at P31-33 and then perfused 24 hours later for immunohistochemistry. (D) The retrograde tracer CTB-555 was administered at P28, and the mice were tested in contextual fear conditioning at P33-34 and subsequently perfused to assess CTB labeling in the lateral entorhinal cortex at P35.

**Fig S2. Effects of LB and Sex on Hippocampal Development in P17 Prepubescent Mice.** (A) Litters were randomized to control (CTL) or limited bedding (LB) conditions at P0 and tissue collected at P17. (B) LB reduced body weight in male and female P17 mice. Main effect of rearing: F (1, 32) = 12.54, P=0.0012, sex: F (1, 32) = 0.010, P= 0.92, interaction: F (1, 32) = 1.16, P= 0.29. (C) representative images of the dorsal hippocampus of P17 CTL and LB pups. Normalized DG vol: F (1, 21) = 11.58, P= 0.0027, sex: F (1, 21) = 1.31, P= 0.26, interaction: F (1, 21) = 0.12, P= 0.73. Normalized CA vol: rearing: rearing: F (1, 25) = 6.93, P= 0.014, sex: F (1, 25) = 0.22, P= 0.63, interaction: F (1, 25) = 0.44, P= 0.51. (D) Effects of rearing and sex on DNA, RNA and protein contents normalized for body weight. Normalized DNA contents: rearing: F (1, 28) = 6.87, P= 0.014, sex: F (1, 28) = 0.14, P= 0.70, interaction: F (1, 28) = 0.091, P= 0.76. Normalized RNA contents: rearing: F (1, 28) = 5.07, P= 0.032, sex: F (1, 28) = 0.084, P= 0.77, interaction: F (1, 28) = 0.017, P= 0.89. Normalized protein contents: rearing: F (1, 28) = 17.12, P= 0.0003, sex: F (1, 28) = 1.91, P= 0.17, interaction: F (1, 28) = 0.156, P= 0.69. Scale bars in B= 500 µm. Error bars represent mean ± SEM. *p< 0.05, **p< 0.01, ***p< 0.001.

**Fig S3. The Top 20 Most Downregulated and Upregulated genes in the P17 hippocampus**. Padj is the adjusted p value after Benjamini-Hochberg correction for multiple comparisons. Since all differentially regulated genes are highly significant (e.g., Padj < 0.05) the genes are ordered based on their effect size.

**Fig S4. LB Impairs Myelination in the SLM of adolescent mice.** (A) Representative confocal images of PDGFRα-positive OPC, CC1-positive mature oligodendrocytes, and MBP in the SLM of adolescent mice. Quantification of number of PDGFRα-positive OPC (B), CC1 mature oligodendrocytes (C) and MBP staining (D) in the SLM. Scale bars in Fig A are 100 microns. N = 5-6 mice per rearing and sex group. Error bars represent mean ± SEM. *p< 0.05.

**Fig S5- Main effect of sex on local volumetric changes in P29 adolescent mice**. Minimal cluster size > 25 voxels, FDR< 0.1, p< 0.005. Areas with significant volume reduction in females are shown in red. Abbreviations: Amy: Amygdala, MO: Motor cortex, OT: Olfactory tubercle, P: pons, RSP: Retrosplenial cortex, SC: Superior colliculus, SS: Somatosensory cortex, VIS: Visual cortex. N= 6 per rearing and sex group.

**Fig S6- CTB Targeting of the Dorsal Hippocampus.** (A) Representative images of CTB staining in the dorsal hippocampus of mice used to assess for retrograde labeling in Fig 9A. (B) Quantification of the signal found no significant effect of rearing F (1, 10) = 0.67, P= 0.43, sex F (1, 10) = 0.03, P= 0.86, or interaction F (1, 10) = 1.073, P= 0.32.
